# Editors’ Prelude to *Microbiome Research Reports*

**DOI:** 10.20517/mrr.2021.01

**Published:** 2021-07-20

**Authors:** Marco Ventura, Douwe van Sinderen, Francesca Turroni, Christian Milani, Jose Munoz, Dirk Haller, R. Paul Ross, Maria Carmen Collado, Emma Allen-Vercoe, Daniele Del Rio, Eric Altermann, Takane Katayama, Erwin G. Zoetendal, Clara Belzer, Pedro Mena, Sin-Hyeog Im, Miguel Gueimonde, Abelardo Margolles, Lorena Ruiz, Christophe Lacroix, Catherine Stanton, Giovani Barbara, Seppo Saminen, Karen P. Scott, Rodolphe Barrangou, Francesca Bottacini, Maria L. Marco

**Affiliations:** ^1^Laboratory of Probiogenomics, Department of Chemistry, Life Sciences and Environmental Sustainability, University of Parma, Parma 43126, Italy.; ^2^Microbiome Research Hub, University of Parma, Parma 43126, Italy.; ^3^School of Microbiology & APC Microbiome Ireland, University College Cork, Cork Co. Cork, Ireland.; ^4^Applied Sciences Department, Northumbria University, Newcastle Upon Tyne NE1 7RU, UK.; ^5^Chair of Nutrition and Immunology, ZIEL - Institute for Food & Health, Freising, Technical University of Munich, Munich 80807, Germany.; ^6^APC Microbiome Ireland, University College, Cork Co. Cork, Ireland.; ^7^Institute of Agrochemistry and Food Technology-National Research Council (IATA-CSIC), Valencia 46007, Spain.; ^8^Department of Molecular and Cellular Biology, University of Guelph, Guelph, Ontario P0R 1A0, Canada.; ^9^Department of Food & Drugs, University of Parma, Parma 43126, Italy.; ^10^AgResearch, Hopkirk Research Centre, Palmerston North 4410, New Zealand.; ^11^Riddet Institute, Massey University, Palmerston North 4410, New Zealand.; ^12^Graduate School of Biostudies, Kyoto University, Kyoto 600-8586, Japan.; ^13^Laboratory of Microbiology, Wageningen University, Wageningen 6700, The Netherlands.; ^14^Department of Life Sciences, Pohang University of Science and Technology (POSTECH), Pohang 999007, South Korea.; ^15^Department of Microbiology and Biochemistry, Institute of Dairy Products of Asturias (IPLA-CSIC), Villaviciosa 33300, Spain.; ^16^Laboratory of Food Biotechnology, Department of Health Sciences and Technology, ETH Zurich, Zürich 8092, Switzerland.; ^17^Teagasc Moorepark Food Research Centre & APC Microbiome Ireland, Cork Co. Cork, Ireland.; ^18^Chair of Internal Medicine, Department of Medical and Surgical Sciences, University of Bologna, Bologna 40121, Italy.; ^19^Functional Foods Forum, Faculty of Medicine, University of Turku, Turku 20100, Finland.; ^20^Gut Health, Rowett Institute, University of Aberdeen, Aberdeen, Scotland AB53, UK.; ^21^Department of Food, Bioprocessing and Nutrition Sciences, North Carolina State University, Raleigh 25911, USA.; ^22^Department of Biological Sciences, Munster Technological University, Bishopstown, Cork Co. Cork, Ireland.; ^23^Department of Food Science and Technology, University of California, Davis 95616, USA.

It is our sincere pleasure to introduce a new scientific journal named *Microbiome Research Reports* (acronym *MRR*), born out of an ambitious initiative from the Editorial Board of this new journal. Our motivation to initiate a new journal on microbiome research was driven by the importance and impact of the microbiome for human and planetary health, with related research interest and effort driven by the scientific community on the subject. In fact, research findings on this subject represent a Copernican Revolution influencing all research branches of the Life Sciences. For example, vast efforts are currently invested in elucidating potential links between microbiome and disease, which could lead to the discovery of microbial biomarkers for novel therapeutic and preventative strategies. We feel that it is very timely to launch a new journal focusing on Microbiome studies in humans and other animals, i.e., both wild- and domesticated- animals, being convinced that it will be a platform for the dissemination of microbiome discoveries.

The aim of *Microbiome Research Reports* is to publish high-quality studies focusing on the analysis of host-associated microbiota communities as well as on microbe-microbe interactions and microbe-host cross-talk. *Microbiome Research Reports* will be interested in publishing studies that investigate the molecular mechanisms underpinning microbe-host and microbe-microbe interactions as well as the microbial/host metabolites, enzymes, extracellular vesicles, cell surface polysaccharides, and other molecules involved in these mechanisms. Notably, these studies must be hypothesis-driven. In fact, a plethora of descriptive studies is available that provide information on the composition of the microbiota residing in different bodily regions of (healthy and diseased) human beings or other animals, although our knowledge on the functional roles played by these microorganisms and the underlying mechanisms involved is still limited. The latter will represent one of the main interests of *Microbiome Research Reports*. Specific interests of *MRR* include:

• The structure, activities and communal behavior of microbial communities;

• Microbial community diversity, genetics and evolutionary processes;

• Microbial symbioses, microbial interactions;

• Microbial metabolic and structural diversity;

• Microbial physiology, growth and survival;

• Microbes and surfaces, adhesion and biofouling;

• Modelling, theory development and new analysis pipelines;

• Viromes/phageome;

• Antimicrobials and competition in microbiomes;

• Fungal communities (mycobiomes);

• Exploration of microbial dark matter;

• Culturomics approaches applied to microbiomes;

• Metabolomics and proteomic approaches applied to microbiomes;

• Metatranscriptomics applied to microbiomes;

• New technological developments in the study of composition and activities of microbial communities, as-yet uncultured microorganisms;

• Gnotobiotic studies;

• Development of minimal microbial consortia and synthetic communities for functional microbe-host studies;

• Probiotics, prebiotics, synbiotics and postbiotics;

• Microbial-derived metabolites originating from food/feed and playing a role on host physiology;

• Effect of microbiota modulation in gastrointestinal disorders;

• Effect of microbiota modulation in metabolic disorders;

• Novel treatments and clinical trials;

• Impact of dietary components on the microbiota, and inter-individual variation in responses;

• Host-microbiome interaction in immune response.

Microbiome studies involving multi-omics approaches and those providing insights into the molecular mechanisms and metabolic pathways behind those mechanisms used by microbes are very welcome by *Microbiome Research Reports*.

All submissions received by *Microbiome Research Reports* will undergo a rigorous and fair peer-review process which is directed by an Editorial Board that is composed by key scientists across various areas of microbiome research. The Inaugural Issue of our journal will be officially launched in the first quarter of 2022. The accepted manuscript will be online released in advance in the following months.

Our ambition is to become one of the top journals on Microbiome research within a period of three years, and we gratefully take this opportunity to invite you to submit your premium scientific studies to *Microbiome Research Reports*.

## DECLARATIONS

### Copyright

© The Author(s) 2021.

